# Retraction: Genetic Testing for Successful Cancer Treatment

**DOI:** 10.7759/cureus.r202

**Published:** 2025-11-19

**Authors:** Desh Nidhi Singh, Sushma Daripelli, Mohamed Osman Elamin Bushara, Georgiy Georgievich Polevoy, Muthu Prasanna

**Affiliations:** 1 Microbiology, Rama Medical College Hospital & Research Centre, Kanpur, IND; 2 Anatomy, Government Medical College (GMC) Jangaon, Jangaon, IND; 3 Anatomy, Gandhi Medical College, Hyderabad, IND; 4 Anatomy, All India Institute of Medical Sciences, Bibinagar, Bibinagar, IND; 5 Health Promotion & Health Education, Umm Al-Qura University, Makkah, SAU; 6 Physical Education, Moscow Polytechnic University, Moscow, RUS; 7 Pharmaceutics and Pharmaceutical Biotechnology, Surya School of Pharmacy, Surya Group of Educational Institutions, Villupuram, IND

The Editors-in-Chief have retracted this article. An investigation by the journal found evidence of authorship manipulation. The Editors-in-Chief therefore no longer have confidence in the provenance and reliability of the article contents. The authors disagree with the decision to retract.

